# Development of an impact evaluation framework and planning tool for field epidemiology training programs

**DOI:** 10.1186/s12960-025-00974-9

**Published:** 2025-04-14

**Authors:** James A. Flint, Michelle Jack, David Jack, Rachel Hammersley-Mather, David N. Durrheim, Martyn D. Kirk, Tambri Housen

**Affiliations:** 1 The University of Newcastle, Newcastle, Australia; 2 The University of Sydney, Sydney, Australia; 3 Social Impact Story, Casuarina, Australia; 4 Australian National University, Canberra, Australia

**Keywords:** Field epidemiology training, FETP, Impact evaluation, Evaluation framework

## Abstract

**Background:**

Despite the growth and diversification of Field Epidemiology Training Programs (FETPs) globally, there are few published evaluations. Those that have been published largely focus on program processes and outputs, with some including short–medium-term outcomes and very few focusing on sustained impact. This paper describes the process of developing an FETP impact evaluation framework and FETP impact evaluation planning tool to facilitate FETP impact evaluations. The impact framework was developed to be simple, flexible and efficient.

**Methods:**

A theory of change process for an FETP in Papua New Guinea formed the basis of the impact evaluation framework. With support from independent impact evaluation experts, the framework was developed using an iterative approach. A review of the literature and technical input from FETP representatives underpinned its development. A simple planning tool was developed to help operationalise the impact framework.

**Results:**

The final FETP impact evaluation framework consists of a high-level summary framework and a detailed operational framework. The high-level framework follows the flow of outputs, outcomes and impacts for trainees, graduates, the public health systems, and communities. The detailed FETP Impact Evaluation Framework includes activities, enablers and barriers, and output, outcome and impact indicators. The FETP Impact Evaluation Planning Tool consists of five steps using a theory-based approach.

**Conclusions:**

The long history and global growth of the FETP model suggest success and imply impact, yet few published papers provide necessary backing evidence. There is growing interest across the FETP community and funders to understand the longer-term changes that FETPs contribute to. We developed an impact framework and planning tool specifically designed to support FETP impact evaluation. The framework and tool are intended to be used by FETP staff with no prior evaluation experience. The evaluation approach is intentionally flexible, allowing contextual application and integration with established quantitative and qualitative evaluation methods.

**Supplementary Information:**

The online version contains supplementary material available at 10.1186/s12960-025-00974-9.

## Introduction

There are 98 field epidemiology training programs (FETPs) around the world that train health professionals to collect, analyse, and interpret public health information, using evidence to take action that reduces public health risk and saves lives [1]. The guiding principle behind FETPs is *learning by doing*; this approach sees trainees spending the majority of their time in the field applying skills acquired during face-to-face workshops [2]. The FETP model was developed by the United States of America Centers for Disease Control and Prevention (CDC) in 1951 and has grown globally, with many countries establishing programs [1, 3]. Over time, programs have moved towards cultural and contextual adaptation to improve local relevance, ownership, and long-term impact. Curricula have been rewritten to incorporate preferred learning styles and updated with country-specific examples and case studies. Today, there are Frontline, Intermediate and Advanced programs, as well as laboratory, non-communicable disease, One Health and veterinary-focused programs [1]. There is also diversity in where programs are housed; they can be in departments of health, National Public Health Institutes, and tertiary institutions [4]. Understanding the outcomes and impacts of these different FETP programs will provide useful learnings for funders, FETP staff, governments and the wider FETP community.

Despite the apparent success, growth, and diversification of FETPs globally, programs rarely conduct or publish evaluations. Where evaluations are published, they largely focus on process or output indicators, such as the number of outbreaks investigated or surveillance systems evaluated [3, 5–12]. Few evaluations have concentrated on program outcomes [6, 13–16], and even fewer on program impacts [17, 18]. As the key driver behind FETPs is to improve the health of populations by strengthening local capability to detect, investigate and respond to public health threats, an understanding of how FETPs contribute to these is essential. This is especially pertinent in the aftermath of the COVID-19 pandemic, with calls for a massive scaling up of FETPs to improve the global health architecture in preparation for the next pandemic [19]. Capturing the outcomes and impacts of FETPs is necessary to provide the evidence base necessary for strategically strengthening, replicating and scaling national programs.

FETPs are resource-intensive training programs requiring government support and engagement, strong program coordination and management, ongoing evaluation and curriculum development, and continual liaison with field sites and supervisors. Considerable time investments are required from mentors and supervisors, especially during the fieldwork phases when trainees are applying their skills in their workplace. This in-service training model necessitates small cohorts with low mentor-to-trainee ratios resulting in a comparatively high per-capita cost. For governments and funders to continue investing in FETPs, and for programs to be institutionalised within national health systems with dedicated resources allocated, their outcomes and impact must be documented.

Impact is understood and defined in many ways. We consider impact as the long-term, sustained change experienced by beneficiaries of initiatives and activities designed and delivered in response to identified challenges or problems. An impact can be positive or negative, intended or unintended. Impact is the pinnacle of a process often described as a theory of change or program logic model that includes a description of the inputs, outputs and outcomes that contribute to impact. An impact evaluation framework informs an impact measurement plan that may include surveys, interviews, observations, or the use of existing data. Impact measurement allows organisations to assess whether: programs and initiatives are working effectively; scarce resources are being applied in the best way possible; and the program is making any difference in the lives of the intended beneficiaries.

This paper describes the process of developing an FETP impact evaluation framework and FETP impact evaluation planning tool. We piloted the impact evaluation framework and planning tool with the FETP in Papua New Guinea (PNG) and present the PNG case study to illustrate the application of the tool in practice.

## Methods

We adopted a theory-based approach to meet our objective of developing an FETP impact evaluation method that is simple, flexible and efficient. The process consisted of four phases; developing a program theory, conducting a review of the literature, constructing an impact evaluation framework, and developing an impact evaluation planning tool.

### Context

The impact evaluation framework and planning tool were piloted in Papua New Guinea (PNG). The PNG FETP (FETPNG) was designed to meet the country's unique needs [14]. Since 2013, PNG’s 9-month intermediate FETP has graduated over 100 field epidemiologists who work across all 22 provinces. More recently, an advanced/extended FETP (18 months) and a Frontline One Health FETP (3 months) have been introduced. PNG’s National Department of Health manages the program with implementation support from technical partners, including the World Health Organization, the US CDC and Field Epidemiology in Action (University of Newcastle and Hunter New England Health, Australia).

### Theory of change

We facilitated a participatory theory of change workshop in PNG prior to the commencement of the advanced/extended FETP in 2019. The workshop engaged 18 key partners, including the FETP director, FETP staff members, donors, technical partners, and graduates of PNG’s intermediate FETP. The Theory of Change process was based on the Acknowledge Facilitators Source Book [20] and included defining the program vision, identifying long-term outcomes, backward mapping to identify a sequence of pre-conditions and individual outcomes leading to the long-term outcomes, and identifying assumptions. This theory of change process guided the development of the impact framework and assisted in generating an initial list of potential evaluation indicators.

### Literature reviews

We reviewed the literature on the evaluation of FETPs, focusing on publications between 1980 and 2022. Journal titles and abstracts were searched in PubMed [21], Directory of Open Access Journals [22] and Scopus [23] databases using the following criteria: “Field Epidemiology Training” OR “FETP” OR “Applied Epidemiology” OR “Epidemic Intelligence” AND (“evaluation” OR “assess*” OR “review*”). A total of 290 articles were screened, with 17 being selected for final review. Articles were excluded by manual review of abstracts; papers that did not describe an evaluation of an FETP or were not written in the English language were excluded. The literature review is being prepared for publication.

A secondary review of grey literature supplemented the literature review and focused on learnings from impact evaluations of other health professional training programs. The aim was to assess the relevance of global indicators in measuring the impact of health-related work-integrated training programs. The following search strings were used in Google: ‘health professional training impact evaluation’, ‘impact evaluation health professional education’, ‘health professional training monitoring and evaluation’, ‘how do you evaluate educational programmes in the health professions’, ‘how do you evaluate health professional education’, ‘how do you evaluate the impact of training’, ‘what is impact evaluation in health promotion’. Based on existing knowledge of the Sustainable Development Goals (SDGs) and indicators banks, the following documents and databases were reviewed: SDG Indicator Framework updated March 2020 [24], IRIS + Thematic Taxonomy updated June 2021 [25], IRIS SDGs Alignment May 2019 [26], WHO Global Health Observatory indicators [27], IHR Core Capacity Monitoring Framework 2018 [28], Joint External Evaluation Tool [29], IHR State Party Self-Assessment Annual Report 2021 [30], and WHO benchmarks for IHR capacities. [31]

### Framework development

Independent impact evaluation experts were engaged to guide the development of the FETP impact framework. The team reviewed PNG’s FETP program documentation and outputs from the theory of change. A total of 33 documents and/or websites were identified, reviewed and summarised, including program information, conceptual models, evaluation indicators and tools and survey results. The team spoke with FETP staff, advisors, and key stakeholders to understand how the program was perceived and how the program was currently measuring and evaluating success. Based on document reviews and interviews, the team drafted an evaluation framework, mapping program enablers, barriers, inputs, outputs, outcomes, and impacts.

Through an iterative process, we developed an FETP impact evaluation framework. Two versions were produced: a high-level summary focusing on the structure and flow of the framework and a detailed framework including enablers, barriers and a suite of potential evaluation indicators. The potential indicators were initially informed by PNG’s FETP theory change and subsequently reviewed and collapsed into indicators that could be applied to FETPs more generally. We sought expert technical input on the draft framework during a half-day impact evaluation workshop at the 11th Training Programs in Epidemiology and Public Health Interventions Network (TEPHINET) Global Conference held in Panama on September 4, 2022. Following a series of presentations outlining the framework and stepping through its application, participants worked in four groups to review and provide feedback on both the high-level and detailed impact frameworks. The groups explicitly reflected on the sequence presented in the high-level framework that outlined the theoretical basis of how an FETP results in change. When reviewing the detailed framework, the four groups focused on assessing, editing, deleting, and adding to the list of potential evaluation indicators and performance measures. A total of 27 individuals, representing FETPs from 12 low-, middle-, and high-income countries, participated in the review. Feedback was captured and referenced in a revised draft of the framework. The framework was also independently reviewed by FETP staff of the Canadian Field Epidemiology Training program, with additional indicators added.

### Development of the planning tool

We subsequently developed a stepwise planning guide and accompanying tool to operationalise the impact framework. The planning tool was guided by prior evaluation experience as well as the current impact evaluation planning activities in PNG.

## Results

The final FETP impact evaluation framework consists of a high-level summary framework and a detailed operational framework.

### High-level FETP impact framework

The high-level framework follows the flow of outputs and outcomes for trainees, graduates, public health systems, and communities, ultimately leading to the desired impact (Table 1).

**Table 1:** High-level field epidemiology training program impact evaluation framework, 2023

Audience	Outputs (product, projects, activities)	Outcomes (short- and medium-term effects of program)	IMPACT (long-term effects of program)
Trainees	*Outputs during the training program* Trainees participate in competency-based Field Epidemiology Training Program and apply skills and knowledge	*Short term outcomes achieved by trainees during training* Trainees are competent and committed to applying their skills and knowledge in their workplace	
Graduates	*Individual outputs following graduation* Graduates develop and apply skills to strengthen disease surveillance, investigate outbreaks, conduct operational research and share findings through papers, reports and presentations	*Short- and medium-term outcomes by individual graduates* Skilled graduates strengthen public health activities in their workplace and contribute to a community of practice through alumni networks	
Public Health System	*Outputs affecting the wider public health system* Graduates embedded across all levels of the public health system, conducting projects and activities that strengthen public health systems. Graduates become junior FETP faculty	*Short- and medium-term outcomes affecting the wider public health system* Experienced Field Epidemiology workforce contributes to strengthening the public health system through routine application of knowledge and skills. FETP graduates support FETP as trainers and mentors	*Longer-term effects of the program on the wider public health system* Strong public health systems across country. Strong and Sustainable FETP is established
Community	*Outputs that affect the community* Community based public health activities and outreach programs conducted	*Short- and medium-term outcomes affecting the community* Improved access to higher quality public health services addressing priority community needs; community engaged in public health decision making	*Longer-term effects of the program on the community* Improved public health realized through reduced morbidity and mortality from communicable and non-communicable diseases

### Detailed FETP impact evaluation framework

The detailed FETP Impact Evaluation Framework (Annex A) includes activities, enablers and barriers, and output, outcome and impact indicators. The indicators listed are extensive, but not comprehensive. They are designed to provide options for evaluators to consider as they work through the process of planning an impact evaluation. Evaluators should prioritise indicators for inclusion in an impact evaluation based on the key evaluation questions, as well as the overall purpose of the evaluation.

### FETP impact evaluation planning tool

**Fig. 1:**
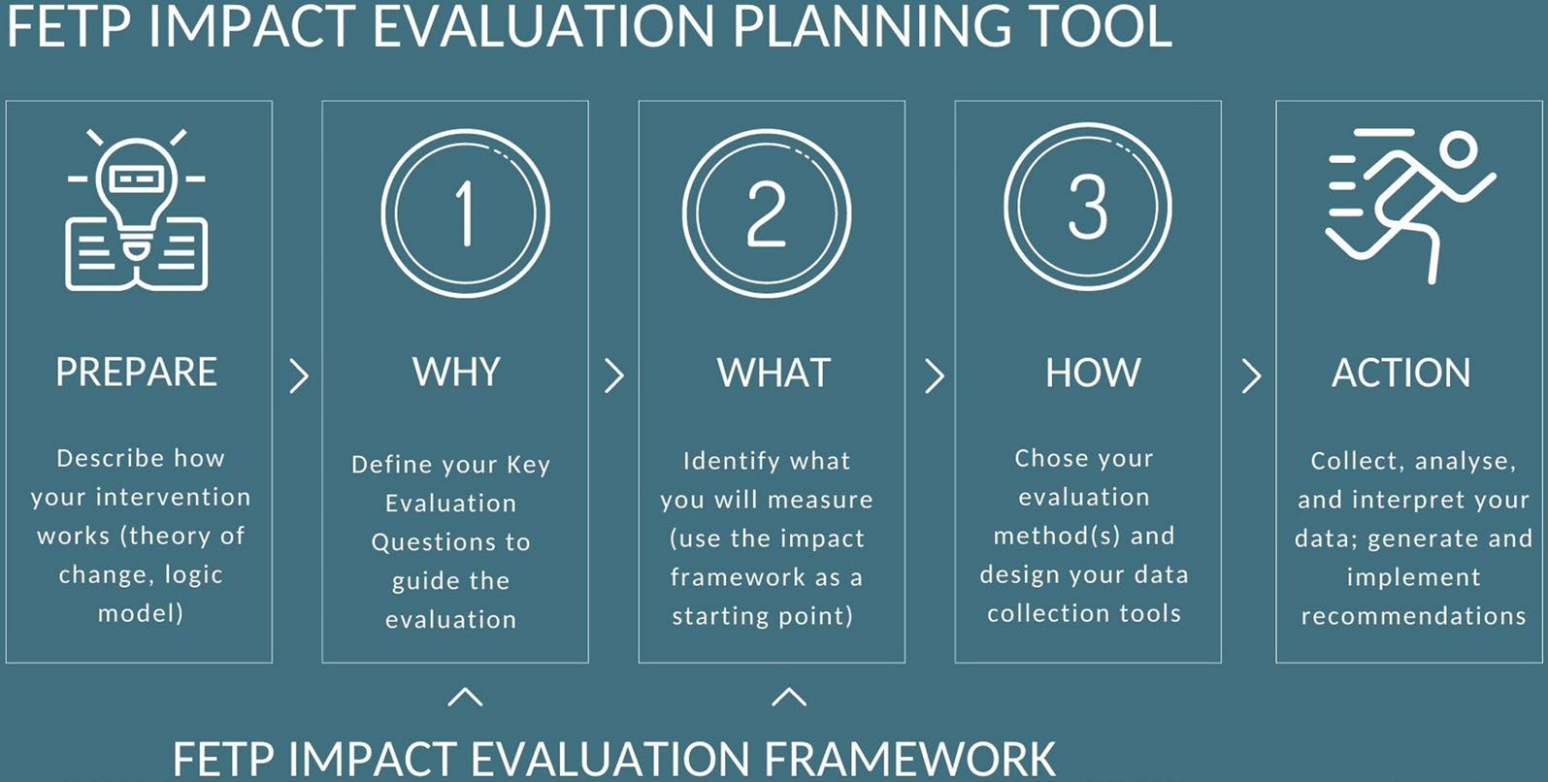
Field epidemiology training program impact evaluation planning tool, 2023

The FETP Impact Evaluation Planning Tool provides a stepwise approach to conducting an impact evaluation using a theory-based approach (Fig. 1). It includes a simple Excel tool to support the prioritisation of indicators and the selection of data collection methods and tools [see Additional file 1].

The steps of the planning tool are outlined below, with examples of their application based on the PNG context.

#### Prepare

This initial step focuses on understanding the program theory of the FETP being evaluated; that is, the explanation of how the FETP is expected to produce results. The program theory is typically summarised in a theory of change diagram or logic model. While the FETP impact evaluation framework is a generic FETP theory of change, it is designed to be used alongside a theory of change or logic model developed specifically for the evaluation of the FETP. An FETP-specific program theory provides additional details unique to the FETP and will be important for guiding the development of key evaluation questions and prioritising indicators for inclusion in the evaluation. Program theory provides the basis for assessing the extent to which the actual results match what was expected. This can be assessed using beneficiary/expert attribution (did participants and/or stakeholders believe the program made a difference), temporality (did the outcomes/impacts occur at a time consistent with the theory), predictions (did participants or sites predicted to achieve best outcomes/impacts do so) or comparative case studies (did the program produce results only when the necessary elements were in place). [32, 33]

**Table:** 

*Example application.* In Papua New Guinea, a program-specific theory of change was developed during a 2-day workshop with key partners as described in the methods section above. A depiction of the resulting theory of change is shown in Annex B. This theory of change served as a basis for the impact evaluation	

#### Why

Defining the evaluation purpose (why) and key evaluation questions (KEQs) is a critical stage of the planning process. Evaluations are summative and/or formative in nature. Summative evaluations are designed to make decisions about whether to continue, replicate or scale a program, while formative evaluations focus on improving the program. FETP evaluations may call for both summative and formative evaluation to meet the needs of funders, program directors and FETP staff.

KEQs should be open-ended questions that are specific enough to focus the evaluation while being broad enough to be broken down into more detailed mid-level evaluation questions to guide data collection. The FETP impact framework, the program-specific theory, and the over-riding purpose of the evaluation all guide the development of the KEQs. KEQs focus on results produced, what has and has not worked, for whom, and in what circumstances.

**Table:** 

*Example application.* The purpose of the evaluation in Papua New Guinea was to guide program improvements and to determine if continued or expanded support of the program is justified. The following KEQs were developed with the FETP staff in Papua New Guinea: 1. What were the key outputs of the FETP? 2. To what extent did the FETP contribute to increased knowledge and skills of trainees and graduates (including enablers and barriers)? 3. To what extent did the FETP trainees and graduates translate knowledge and skills into public health action (including enablers and barriers)? 4. To what extent did the FETP contribute to a health system responsive to public and clinical health needs (including enablers and barriers)? 5. To what extent did the FETP graduates impact public health in the communities they serve (including enablers and barriers)? 6. To what extent did the FETP contribute to the sustainability of Field Epidemiology Training Programs in PNG (including enablers and barriers)? 7. What were the unintended positive and negative consequences of the FETP on trainees and graduates? Mid-level evaluation questions were subsequently developed under each KEQ	

#### What

The next step in the planning process is determining what data and information need to be collected. The detailed FETP impact evaluation framework (Annex A) provides a starting point for the selection of indicators and performance measures. The choice of indicators is narrowed using the KEQs. A final selection of prioritised indicators is based on the strength of alignment to the evaluation questions and the feasibility and practicality of measuring them. The Excel tool developed supports the indicator prioritisation process.

**Table:** 

*Example application.* Using the Excel prioritisation tool, the evaluators for the Papua New Guinea FETP independently prioritised the indicators for inclusion. Prioritisation was based on alignment with the KEQs and the ease at which the data could be collected. After individual prioritisation, evaluators met to compare indicator selection and reach a consensus on the final list of indicators. The prioritisation process was also used to refine the mid-level evaluation questions in an iterative process	

#### How

This step determines the methods to be used and the tools to be developed for the evaluation. It is important to maximise the use of existing data collected by the program, as well as data available from other sources. Existing data sources may include FETP program documents, workshop reports, monitoring data, trainee/graduate databases, surveillance data, published papers, and the like. Inevitably, there will be data gaps requiring primary data collection. Primary data collection options include surveys, interviews, focus groups and observations of trainees, graduates, FETP staff, line managers and/or community members. Evaluators may also choose to use one or more established evaluation models, such as Kirkpatrick’s four levels of evaluation [34], CIPP evaluation model [35], Most Significant Change [36], Outcome Harvesting [37], or the Brinkerhoff Success Case method. [38]

Mixed methods, using both quantitative and qualitative methods, strengthen the evaluation by helping to overcome the inherent weaknesses of each method when used alone. [33] Triangulation of findings from quantitative and qualitative methods also increases the credibility of evaluation findings when information from different data sources converge; that is, there is consistency about the direction of the findings across different data sources [39]. Divergent results, on the other hand, can reveal findings that need further explanation that lead to deeper insight [40]. It is essential to pre-plan how the quantitative and qualitative data will be integrated to answer the KEQs. Several mixed method designs and analysis methods are suitable for impact evaluation, including triangulation design, embedded design, explanatory design, and exploratory design. [39, 41].

**Table:**
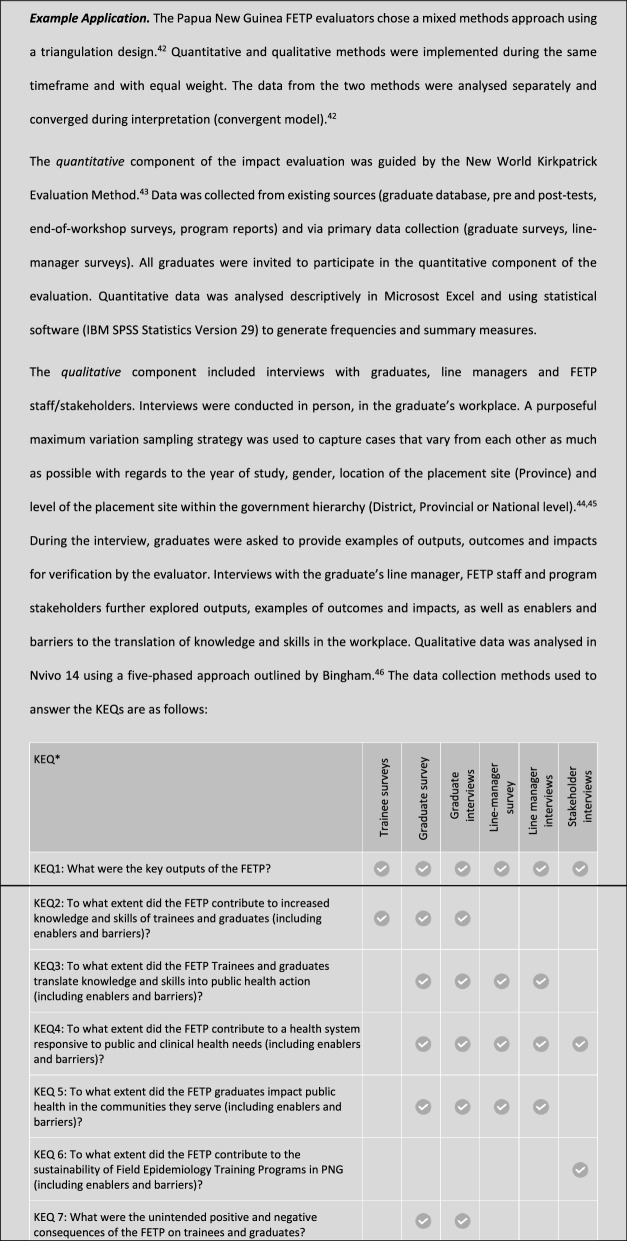

#### Action

The final step includes data collection, analysis and interpretation. The timing of when to collect the data depends, in part, on the evaluation timeframe and available resources. FETPs usually collect programmatic data throughout the training phase, capturing data on outputs and short-term outcomes. As impact evaluation focus on both medium-term outcomes, as well as the longer-term effects of the training, data collection post-graduation is required. Graduates need time to apply their knowledge and skills in the workplace before being assessed.

The frequency of conducting an impact evaluation likewise depends on available resources, as well as evaluation needs. While it is good practice to evaluate every cohort, a full impact evaluation will likely occur only after several cohorts have graduated. For established programs, conducting a full impact evaluation every 5 cohorts is ideal. This timeframe offers enough time to accumulate a sufficiently large sample of graduates while remaining short enough to assess the impact of changes implemented since the previous evaluation. However, for new programs or programs undergoing significant changes, an impact evaluation may be necessary after one or two cohorts.

**Table:** 

*Example application.* In PNG, surveys were administered to participants throughout the training. These include pre- and post-tests and end of the workshop and end-of-program surveys. A post-graduation survey of graduates was administered > 12 months following graduation. Interviews with graduates and line managers were also conducted > 12 months following graduation. Data integration combined findings from qualitative and quantitative data collection in the analysis phase to identify convergent and divergent findings [46]. This technique involved producing a ‘convergence coding matrix’ to display findings emerging from each component of the study. [46, 47]	

## Discussion

The drive for effective FETP impact measurement primarily comes from two sources. First, FETP directors and staff who want to measure impact and effectiveness so they can deliver programs in the best possible way and secure ongoing funding to sustain them. Secondly, governments and funders who increasingly require impact measurement to track and understand the returns on their investments [48]. Although rarely evaluated, FETPs have been designed with impact in mind. It has been stated that for a field epidemiologist, the task is “not complete until results of a study have been conveyed clearly to those who need to know, and an intervention has been implemented to improve the health of the people” [6]. Or as William Foege, former director of the United States Centers for Disease Control and Prevention, once said while addressing the topic of field epidemiologists, “we exhort them to strive not only for academic rigour but also for public health consequence. A difference to be a difference must make a difference” [3]. The resource-intensive ‘learning by doing’ model of FETPs reflects this commitment. Impact evaluation provides the evidence to assess outcomes and enable programs to improve and innovate for greater impact using evidence-based decisions. Developing a culture of evaluation is especially important given the considerable opportunity costs associated with extracting health workers from their workplaces, especially in low-resource settings.

The paucity of FETP evaluation evidence is in the domains of outcome and impact. There are numerous publications highlighting outputs. However, the quantity, and even quality, of outputs does not necessarily equate to service, organisational, or public health impact [17]. Investigation of outbreaks, evaluation of surveillance systems, conducting field projects, publishing papers and presenting at conferences, while useful output indicators, all fail to advance health in any significant way unless they are translated into practical outcomes. Evaluating outcomes and impact is more complex, more time-consuming and resource intensive. The sheer variety and diversity of impact evaluation methods can be daunting, leaving FETP evaluators confused and ill-equipped. In reality, FETP evaluators, who are often graduates of an FETP themselves, are well equipped to undertake evaluation activities due to the nature of their training. The impact framework and planning tool presented above provide a structured approach that can be flexibly applied [49].

A theory-based evaluation approach is well suited to FETP impact evaluation. Theory-based approaches attempt to understand a program's contribution to observed results through a mechanistic or process interpretation of causation rather than determining causation through comparison to a counterfactual. Identifying your contribution and recognising the contribution of others is more realistic than searching for evidence of sole attribution [50]. While impact evaluation aims to look at the longer-term results of the program, decision-makers and funders often need more timely information. This may lead evaluators to focus on proxies for impact, such as career promotion; a proxy for organisation impact [48]. This approach is valid, providing the program theory is sound. Data collected on program *outcomes* are used to assess whether the program is on track to achieve the anticipated *impacts*. These proxy outcomes, or leading indicators*,* suggest critical behaviours are on track to achieve the intended impact and vision of the program.

The debate surrounding the use of standardised frameworks for evaluating program outcomes has been ongoing for a decade in the wider development sector. Advocates for standardisation highlight the benefits of allowing great consistency, summation and aggregation of results across programs and organisations, simplified tool development, and reduced overall costs [48]. At the heart of the debate is the fundamental challenge of measuring and understanding the complexity and diversity of outcomes. The trade-off and limitation of this approach is a reduced ability to compare evaluation findings across FETP programs. To address this lack of standardisation, a Delphi study is planned with international FETP experts to prioritise a set of core indicators for inclusion in FETP impact evaluations. This will enable inter-program comparison of common indicators while retaining the flexibility of the overall evaluation approach.

This framework builds on three guidance documents that address FETP evaluation. The CDC Field Epidemiology Training Program Development Handbook outlines an approach using Kirkpatrick’s four levels of evaluation [51]. The second, a Continuous Quality Improvement Handbook, published by TEPHINET, presents an evaluating framework focusing on program inputs, processes, outputs, outcomes and impact [52]. In total, there are 173 indicators recommended for evaluation; most are input, process, and output indicators, with one related to impact. The third document, also published by the CDC, outlines a scorecard approach covering five domains; competency-based training, public health work/field activities, public health leadership, management, and sustainably [13]. The framework we developed emphasises program theory to focus key evaluation questions and indicator selection and provides flexibility in the selection of methods. Kirkpatrick is one of several suitable methods. Our framework also includes key structural levels where the program is expected to exert influence; trainees, graduates, health systems, and community. The inclusion of structural levels is common practice in the evaluation of capacity-strengthening development programs [53–55]. The framework of Levels and Dimensions that emerged in the mid-1990s indicated that any capacity-strengthening initiative should be examined from all proposed structural levels [55]. The WHO adopts a framework that has five structural levels that take into account the complexity of the public sector: individual, organisational, network, institutional and action environment [54, 55]. Cooke’s evaluation framework for research capacity building includes individual, team, organisation and network. [53]

One of the limitations of our framework is that it does not incorporate an economic component for assessing the social or financial return on investment. We recommend further work to assess the best approach to assessing the cost benefit of FETPs and developing a model specifically in support for FETPs to support.

While the framework and implementation guide provide support for evaluators, considerable investments of time and financial resources will be required to develop the program theory, select methods, develop data collection tools, collect and analyse data, disseminate findings and implement recommendations. If FETPs are to generate the evidence base to assess their impact, programs need to adequately plan and resource evaluation activities. The framework and planning tool presented here serve as a starting point for FETP program evaluators and will undoubtedly be modified and adapted over time. The list of indicators will evolve as programs use the framework for their FETPs and share feedback. As FETPs embark on impact evaluations it is recommended that tools, templates, guides, and lessons learnt are shared within the FETP community as we continue to refine the process of capturing impact.

## Conclusion

Evidence-based decision-making is a basic tenet of FETPs. However, a firm evidence base that demonstrates the FETP model is itself achieving impact is lacking. The long history and global growth of the FETP model suggest success and imply impact, yet few published papers provide the necessary evidence. There is growing interest by many in the global FETP community to address this. We have developed an impact framework and planning tool specifically designed to support FETP impact evaluation. The framework and tool are designed for program FETP staff from any FETP. It is intentionally flexible, allowing contextual application and integration with established quantitative and qualitative evaluation methods.

## Supplementary Information

Additional file 1.

## Data Availability

Not applicable.

## Detailed FETP Impact Evaluation Framework

**Table:**

Audience	Activity	Enablers/barriers *Factors that support or hinder the development of anticipated outputs, outcomes and impacts*	Outputs *Products, projects, or activities which result from the training program* [Trainees: outputs during the training program]	Outcomes *Short-term and medium-term effects of the training program* [Trainees: short-term outcomes achieved by trainees during training]	Impact *Longer term effects produced by the training program* [Trainees: not relevant]
**Trainees**	**Field epidemiology training** providing knowledge and applied skills	**Enablers** • Competency based training • Trainees knowledge & skills at entry to program • Recruiting ‘ideal’ candidates (e.g., personal motivation of trainees) • Key stakeholders support & fund program • Co-designed curriculum • Program and curriculum design based on needs assessment and aligned to local priorities • Local ownership & local leadership of the program • Cultural and contextual adaptation of training materials • Supportive trainees network • Local FETP staff available, skilled and motivated to identify, train and mentor trainees • International experts available to train and support local FETP staff as needed • Skilled trainers with suitable trainer:trainee ratio • Supportive mentors for trainees with suitable mentor:trainee ratio • Sustainable funding source(s) **Barriers** • Poor training facilities or training conditions • Competing work priorities and demands limiting availability of trainees and FETP staff • Diversion of FETP staff and trainees due to outbreak or emergency response • Non-supportive attitude of host organization • Host institution doesn’t fully appreciate role of FETP • Unrealistic expectations regarding the program and role of trainees after graduation • Mentors from ‘outside’ who do not understand local context • Inability of trainees to be deployed into the field	**Trainees participate in competency-based Field Epidemiology Training Program and apply skills and knowledge** **Training Participation** • Gender breakdown of fellows • Number/percentage of fellows enrolled in training by role, position, and workplace location • Number/percentage of fellows linked with public health mentors/supervisors **Training Quality and Relevance** • Number of hours of training in a classroom type setting (including virtual training) • Total length of time in the field/workplace (in weeks) • Number/percentage of fellows reporting enjoyable learning experience • Number/percentage of fellows reporting training relevance to current role • Number/percentage of fellows reporting improved field knowledge and skills in key field epidemiology competencies • Number/percentage of fellows that meet program’s core competencies • Approximate number of hours of direct one-on-one mentoring provided to fellows **Operational Research, Surveillance and Outbreak Investigation** • Number of operational research studies completed by fellows [Number/percentage of fellows completing operational research studies] • Number of surveillance systems evaluated by fellows [Number/percentage of fellows evaluating surveillance systems] • Number of outbreak investigations led by fellows [Number/percentage of fellows leading outbreak investigations] • Number of outbreaks investigations supported by fellows [Number/percentage of fellows supporting outbreak investigations] • Number of national mobilizations involving fellows [Number/percentage of fellows mobilized nationally] • Number of international mobilizations involving fellows [Number/percentage of fellows mobilized internationally] • Number/percentage of deployed fellows who undertake post-deployment debriefs • Number/percentage of fellow mobilizations meeting needs of requestor **Written Products, Presentations and Processes** • Number of epidemiological reports prepared by fellows [Number/percentage of fellows preparing epidemiological reports] • Number of policy briefs written by fellows [Number/percentage of fellows writing policy briefs] • Number of policies updated or developed by fellows [Number/percentage of fellows updating or developing policies] • Number of health program recommendations made by fellows [Number/percentage of fellows making health program recommendations] • Number of papers published with fellows as lead authors [Number/percentage of fellows publishing papers as lead authors] • Number of papers published with fellows as co-authors [Number/percentage of fellows publishing papers as co-authors] • Number of presentations within fellows’ workplace or placement site [Number/percentage of fellows giving presentations in workplace / placement site] • Number of presentations outside of fellows’ workplace/placement site [Number/percentage of fellows giving presentation outside workplace / placement site] • Number of conference abstracts submitted by fellows being accepted [Number/percentage of fellows having conference abstracts accepted] • Number of conference presentations given by fellows [Number/percentage of fellows giving conference presentations] • Number of work-related processes or procedures updated or developed by fellows [Number/percentage of fellows updating or developing work-related processes or procedures]	**Trainees are competent and committed to applying their skills and knowledge in their workplace** **Trained Field Epidemiologists** • Fellows demonstrate the application of field epi competencies throughout the training • Fellows are provided quality mentoring throughout training program • Fellows are confident in applying their knowledge and skills in their workplace / placement site **Competent Field Epidemiologists** • Fellows undertake field projects, such as surveillance evaluations, operational research, or interventions • Fellows contribute to improvements in work-related processes or procedures at their workplace/placements site • Fellows improve a disease surveillance system(s) • Fellows analyse and interpret surveillance data to inform decision making • Fellows investigate outbreaks by appropriately following the steps of an outbreak investigation • Fellows implement or support the implementation of appropriate control measures during outbreak investigations • Fellows report unintended positive and/or negative consequences of FETP	

**Table:**

Audience	Activity	Enablers/barriers *Factors that support or hinder the development of anticipated outputs, outcomes and impacts*	Outputs *Products, projects, or activities which result from the training program* [Graduates: individual outputs following graduation]	Outcomes *Short-term and medium-term effects of the training program* [Graduates: short term individual outcomes achieved by graduates]	Impact *Longer term effects produced by the training program* [Graduates: not relevant]
**Graduates**	**FETP graduates contributing** across different levels of the public health system​	**Enablers** • Sustained mentorship and supervision • Strong networks • Supportive workplace • Motivated graduates • Delineated role of field epidemiologist in health system; clear career pathway • Ongoing training / professional development opportunities for graduates **Barriers** • Trainees’ line managers do not understand/appreciate the role of a field epi • Trainees job/responsibilities following graduation remain unchanged • Trainees leave substantive jobs after graduation • No employment opportunities for trainees following graduation • No promotional opportunities following graduation • Compensation not competitive compared to clinical roles • Lack of advocacy on how to utilize skills of FETP graduates	**Graduates develop and apply skills to strengthen disease surveillance, investigate outbreaks, conduct operational research, and share findings through papers, reports, and presentations** **Graduates** • Gender breakdown of graduates • Number/percentage of graduates completing FETP training by role, position and workplace location • Number/percentage of graduates undertaking continuing education/professional development training • Number/percentage of graduates completing higher level training (course name and level, e.g., leadership training, Master of Public Health, PhD, etc.) • Number/percentage of graduates given additional responsibilities in their employment because of their field epidemiology training (describe additional responsibilities) • Number/percentage of graduates receiving a promotion within 12 months of graduating **Operational Research, Surveillance and Outbreak Investigation** • Number of operational research studies completed by graduates [Number/percentage of graduates completing operational research studies] • Number of surveillance systems evaluated by graduates [Number/percentage of graduates evaluating surveillance systems] • Number of outbreak investigations led by graduates [Number/percentage of graduates leading outbreak investigations] • Number of outbreaks investigations supported by graduates [Number/percentage of graduates supporting outbreak investigations] • Number of national mobilizations involving graduates [Number/percentage of graduates mobilized nationally] • Number of international mobilizations involving graduates [Number/percentage of graduates mobilized internationally] • Number/percentage of deployed graduates who undertake post-deployment debriefs • Number/percentage of graduate mobilizations that meet the needs of requestor • Number/percentage of graduates that feel confident to respond to emerging issues in public health • Number/percentage of graduates supporting an Incident Management System (IMS) during a public health emergency response **Written products, Presentations and Processes** • Number of epidemiological reports prepared by graduates [Number/percentage of graduates preparing epidemiological reports] • Number of policy briefs written by graduates [Number/percentage of graduates writing policy briefs] • Number of policies updated or developed by graduates [Number/percentage of graduates updating or developing policies] • Number of health program recommendations made by graduates [Number/percentage of graduates making health program recommendations] • Number of papers published with graduates as lead authors [Number/percentage of graduates publishing papers as lead authors] • Number of papers published with graduates as co-authors [Number/percentage of graduates publishing papers as co-authors] • Number of presentations within graduates’ workplace [Number/percentage of graduates giving presentations in workplace] • Number of presentations outside of graduates’ workplace [Number/percentage of graduates giving presentation outside workplace] • Number of conference abstracts submitted by graduates being accepted [Number/percentage of graduates having conference abstracts accepted] • Number of conference presentations given by graduates [Number/percentage of graduates giving conference presentations] • Number of work-related processes or procedures updated or developed by graduates [Number/percentage of graduates updating or developing work-related processes or procedures]	**Skilled graduates strengthen public health activities in their workplace and contribute to a community of practice through alumni networks** **Skilled Field Epi workforce** • Graduates employed in positions where field epi knowledge and skills are required • Graduates provided opportunities to apply skills and knowledge in the workplace • Graduates confident in applying knowledge and skills in the workplace • Graduates improve the overall quality of their work • Graduates progress in their careers as a result of their graduation from an FETP **Contributing Field Epi graduates** • Graduates undertake field projects, such as surveillance evaluations, operational research, or interventions • Graduates improve disease surveillance systems • Graduates routinely analyse and interpret surveillance data to inform decision making • Graduates investigate outbreaks by appropriately following the steps of an outbreak investigation • Graduates implement or support the implementation of appropriate control measures during outbreak investigations • Graduates use evidence-based decision-making processes in the workplace • Graduates are developing or contributing to the development of public health policy • Graduates are introducing improved ways of delivering public health programs • Graduates contribute to improvements in work-related processes or procedures at their workplace • Graduates are recognized, utilized and have influence in the workplace • Graduates transfer field epidemiology knowledge and skills to others through training and mentoring • Graduates apply field epidemiology knowledge and skills to other fields • Graduates contribute to raising the profile of field epidemiology, surveillance and/or public health • Graduates report unintended positive and/or negative consequences of FETP **Networks and Partnerships** • Graduates actively engage with and contribute to an FETP alumni network • Graduates develop and engage in networks and partnership to improve public health practice • Graduates share field epidemiology learnings and good practices with public health colleagues	

**Table:**

Audience	Activity	Enablers/barriers *Factors that support or hinder the development of anticipated outputs, outcomes and impacts*	Outputs Products, projects or activities which result from the training program [Public health system: outputs that affect the public health system]	Outcomes Short-term and medium-term effects of the training program outputs [Public health system: short-term and medium-effects on the public health system]	Impact Longer term positive and negative, primary and secondary long-term effects produced by the training program, directly or indirectly, intended or unintended [Public health system: longer term effects of the program on the public health system]
**Public Health System**	**Health systems strengthening** through application of field epidemiology skills	**Enablers** • Support from alumni network • Sustained mentorship and supervision • Support from managers & other government stakeholders • Adequate infrastructure, resources and supplies to deliver public health programs • Graduates are in decision-making roles **Barriers** • Lack of or weak public health systems • Lack of leadership in public health • Complicated processes that restrict public health action	**Graduates embedded across all levels of the public health system, conducting projects and activities that strengthen public health systems. Graduates become junior FETP staff** **Field Epidemiology Workforce** • Number/percentage of graduates placed across different tiers of health system (e.g., district, provincial, national levels) • Number/percentage of [districts / provinces] in country with graduates • Number/percentage of graduates employed by a government institution • Number/percentage of graduates employed by a non-governmental organization • Number/percentage of graduates employed by an academic institution • Number/percentage of graduates employed in epidemiology or applied public health related positions • Number/percentage of graduates who are members of national, regional or international public health committees or working groups Support for Surveillance and Outbreak Response • Number of disease surveillance systems strengthened by fellows and graduates • Number of outbreak response systems and practices strengthened by fellows and graduates • Number/percentage of outbreaks detected from disease surveillance systems • Number/percentage of communicable disease outbreaks where an investigation commenced within 24, 48 and 72 h Support for Health Systems • Number of evidence-based recommendations implemented by fellows and graduates [Number/percentage of fellows and graduates implementing evidence-based recommendations] • Number of policy recommendations implemented by fellows and graduates [Number/percentage of fellows and graduates implementing policy recommendations] • Number of health program recommendations implemented by fellows and graduates [Number/percentage of fellows and graduates implementing health program recommendations] • Number of work-related processes or procedures implemented by fellows an graduates [Number/percentage of fellows and graduates implementing work-related processes or procedures] FETP Support • Number/percentage of FETP trainers and mentors who are graduates of the program • Number/percentage of FETP staff (e.g., Director, Convenor) who are graduates of the program • FETP is recognised in official governmental/institutional planning documents • FETP steering committee is established and functional • Number/percentage of FETP positions fully staffed • Percentage of FETP activities funded by national government • FETP accredited by TEPHINET	**Field Epidemiology workforce contributes to strengthening the public health system through routine application of knowledge and skills. FETP graduates support FETP as trainers and mentors** **Influential Field Epi workforce** • Established career pathway for graduates • Graduates are in public health leadership roles in governmental departments and non-governmental organizations • Key surveillance and disease control positions at all tiers of government are occupied by FETP graduates • Skills of graduates are maintained and continually applied • Decision makers confident in and engaged with FETP graduates and the services they provide • Graduates develop and deliver field epidemiology related training activities to workplace colleagues • Graduates are public health influencers in their workplace and the communities/populations they serve **Health Systems Strengthened** • Graduates routinely conduct field projects/operational research to understand and address key public health challenges • Graduates routinely design and implement public health interventions to address public health challenges • Graduates effectively engage with communities when planning and delivering public health programs • Decision makers utilize the evidence generated by graduates to improve public health programming • Graduates are routinely using evidence-based decision making to inform guidelines, policy & programmatic activities • Graduates are driving innovation and service improvements Stronger Surveillance and Outbreak Response • Graduates contribute to improved surveillance resulting in improved public health programming • Graduates contribute to improved surveillance resulting in improved outbreak detection and response • Graduates provide a response-ready workforce for outbreak and public health emergency response activities nationally and/or internationally • Graduates effectively engage with communities/general public when investigating outbreaks and responding to public health threats Graduate Support for FETP(s) • Established pathway for FETP graduates to become FETP trainers, mentors, and program staff • Graduates contribute to the national FETP(s) as trainers, mentors, and program staff	**Strong public health systems across PNG. Strong and Sustainable FETP is established** **Strong Health Systems** • FETP faculty and graduates contribute to the generation of public health intelligence and evidence-based recommendations to improve public health • FETP faculty and graduates contribute to an evidence-based decision-making culture that drives public health programming and practice • FETP faculty and graduates contribute to the development of evidence based public health policies and practices that are accepted, resourced, and implemented • FETP faculty and graduates contribute to strong disease surveillance systems that guide public health programming and consistently supports the early detection & response to public health threats • FETP faculty and graduates are influential in advocating for political and financial support for public health at all levels of the health system • FETP faculty and graduates contribute to a culture of respectful community engagement when designing and delivering public health programs and response activities **Sustainable FETP(s)** • FETP is institutionalized, adequately funded and nationally recognised as an important public health workforce development program • FETP is run by national staff without the need for external support • FETP program is influential across public health networks nationally and internationally

**Table:**

Audience	Activity	Enablers/barriers *Factors that support or hinder the development of anticipated outputs, outcomes and impacts*	Outputs Products, projects or activities which result from the training program [Community: outputs that affect the community/general public]	Outcomes Short-term and medium-term effects of the training program outputs [Community: short term outcomes of the program affecting the community/general public]	Impact Longer term positive and negative, primary and secondary long-term effects produced by the training program, directly or indirectly, intended or unintended [Community: longer term effects of the program on the community/general public]
**Community/General Public**	​**Responsive and Effective Health System** providing health services improving the health of the public	**Enablers** • Links between primary health services and community-based individuals and organizations • Resources available to permit community/population-based programming • Time spent investing in relationships, including providing data feedback to community/general public • Links to local community/cultural structures • Continued contact with community/general public • Community engagement and establishment of trust **Barriers** • Limited access to remote communities • Poor or no connectivity with remote communities • Lack of feedback to community/general public	**Community/population based public health activities and outreach programs conducted** • Number of new or strengthened community/population based public health programs conducted or coordinated by fellows or graduates • Number of community/general public engagement activities conducted by fellows or graduates • Number of community/general public awareness / training activities conducted by fellows or graduates • Number of public health events notified to by the community/general public	**Improved access to higher quality public health services addressing priority community/general public needs; community/general public engaged in public health decision making** • Graduates contribute to improved access to public health services • Graduates contribute to improved quality of public health services • Graduates contribute to improve public health key performance indicators (KPIs), such as vaccine coverage, supervised deliveries, etc • Graduates contribute to improved community/general public engagement resulting in community involvement in public health decision making process affecting them • Graduates contribute to improved health literacy within community/general public	**Improved public health realized through reduced morbidity and mortality to communicable and non-communicable diseases** • FETP faculty and graduates contribute to reduced outbreak related morbidity and mortality through timely and effective outbreak response activities • FETP faculty and graduates contribute to reduced morbidity and mortality through improved access to and provision of public health services • FETP faculty and graduates contribute to reduced morbidity and mortality through improved public health program design and delivery

### Sources of enablers, barriers and indicators listed in detailed Impact evaluation framework

1. Cancedda C, Farmer PE, Kerry V, et al. *Maximizing the impact of training initiatives for health professionals in low-income countries: frameworks, challenges, and best practices*. 2015;12(6):e1001840.
2. Flint JA. *Training Evaluation Theory (*https://www.fieldepiinaction.com/resources-blog/blog-post-title-four-k877j-ykxrf-wd7k3-jczgg-9pbew-t7y7z-8e87g-fh55t-495a3-ytpk7*)*
3. Flint JA, Taylor J, Housen T, et al. Involvement and readiness of trainees from Papua New Guineas Field Epidemiology Training Programme in the COVID-19 response, 2020–2021*. 2023. WSPAR, in press*
4. Field Epidemiology in Action. *Field Epidemiology Training PNG Conceptual Model. 2020 (unpublished diagram)*
5. Flint JA. Measuring the impact of Field Epidemiology Training Programs in Papua New Guinea and Solomon Islands using a mixed methods theory-based approach*.* 2020 (unpublished *Research PhD Proposal*).
6. Field Epidemiology in Action*. Accelerating the development of evidence based policy and practice (ADEPPt) in Papua New Guinea; Thoery of Change. (unpublished report)*
7. Field Epidemiology in Action*. Advanced Field Epidemiology Training of PNG: evaluation indicators. (unpublished docment)*
8. TEPHINET; Input from FETP particpants at Global TEPHINET Conference (impact evaluation interactive learning session), September 2022
9. Canadian Field Epidemiology Training Program
10. World Health Organization. *IHR State Party Self-Assessment Annual Report (SPAR).*
11. World Health Organization.* Joint External Evaluation.*
12. World Health Organisation. *WHO benchmarks for International health regulations (IHR) capacities.* Geneva2019.
13. Flint JA. *Evaluating the Impact of Field Epidemiology Training Programs (*https://www.fieldepiinaction.com/resources-blog/blog-post-title-four-k877j-ykxrf-wd7k3-jczgg-9pbew-t7y7z-8e87g-fh55t-495a3-ytpk7-mdetm*)*
14. Dey P, Brown J, Sandars J, Young Y, Ruggles R, Bracebridge SJE. The United Kingdom field epidemiology training programme: meeting programme objectives. 2019;24(36):1,900,013.
15. Dick VR, Masters AE, McConnon PJ, Engel JP, Underwood VN, Harrison RJJAJoPM. The CDC/Council of state and territorial epidemiologists applied epidemiology traineeship program: evaluation of the first 9 years. 2014;47(5):S376-S382.
16. Dannenberg ALJPcd. Peer reviewed: effectiveness of health impact assessments: a synthesis of data from five impact evaluation reports. 2016;13.
17. Field Epidemiology in Action. Kirkpatrick’s Four Levels of Evaluation; presentation to TEPHINET Regional Conference of the Amercias. 2022 (https://static1.squarespace.com/static/5fb4723e225bcb20d28f0f76/t/616f3ad30017e41b69a8d228/1634679514988/TEPHINET+Kirkpatrick.pdf)
18. Bhatnagar T, Gupte MD, Hutin YJ, et al. Seven years of the field epidemiology training programme (FETP) at Chennai, Tamil Nadu, India: an internal evaluation. 2012;10(1):1–7.
19. Kilbourne AM, Elwy AR, Sales AE, Atkins DJMC. Accelerating research impact in a learning health care system: VA’s quality enhancement research initiative in the choice act era. 2017;55(7 Suppl 1):S4.
20. United Nations *Sustainable Development Goals.*

## Appendix B

Theory of Change summary diagram for the advanced Field Epidemiology Training Program of Papua New Guinea.
